# Sex differences in residual somatic symptoms in patients with first-episode depression after acute-phase treatment

**DOI:** 10.1186/s12888-023-04612-3

**Published:** 2023-02-22

**Authors:** Jingjing Shi, Xiaohong Wang, Na Zhao, Chuanyi Kang, Liying Yang, Yue Zheng, Jiacheng Liu, Lei Feng, Xuequan Zhu, Caina Ma, Wenyuan Wu, Gang Wang, Jian Hu

**Affiliations:** 1 Department of Psychiatry, The First Affiliated Hospital of Harbin Medical University, 23 Youzheng Street, Nangang DistrictHeilongjiang Province, Harbin, 150001 China; 2 Peking University Institute of Mental Health (Sixth Hospital), Beijing, China; 3 National Clinical Research Center for Mental Disorders & NHC Key Laboratory of Mental Health, Peking University, Beijing, China; 4 The National Clinical Research Center for Mental Disorders & Beijing Key Laboratory of Mental Disorders & Beijing Anding Hospital, Capital Medical University, Beijing, China; 5 Harbin First Specialized Hospital, Heilongjiang Province, Harbin, China; 6 Department of Psychiatry, Tongji Hospital of Tongji University, Shanghai, China

**Keywords:** Depression, Sex differences, Residual somatic symptoms, Acute phase treatment, Quality of life

## Abstract

**Background:**

Residual somatic symptoms (RSS) are common in depressed patients, predicting treatment effectiveness. However, sex differences in RSS have received little systematic study. This study was conducted to compare sex differences of RSS in patients with first-episode depression (FED).

**Methods:**

Nine hundred eighty-two patients with FED were selected and treated for 8 to 12 weeks. We evaluated the subjects' socio-demographic characteristics and residual depressive symptoms. Using the Patient Health Questionnaire-15 (PHQ-15) scale to assess residual somatic symptoms, the Sheehan Disability Scale (SDS) for the assessment of patients' function, the Quality of Life Enjoyment and Satisfaction Questionnaire-Short Form (Q-LES-Q-SF) for quality of life.

**Results:**

The incidence of RSS with FED was 46.4%. For patients with residual symptoms, the age and age of onset in females were higher than males, but males had more years of education than females. The degree of "stomach pain" in females was more severe than in males, while "trouble sleeping" in males was more severe than that in females. Multiple regression analysis showed that the total Q-LES-Q-SF score was an independent influencing factor of RSS in both males and females, while the total SDS score only affected female RSS.

**Conclusions:**

The prevalence of RSS in FED after acute-phase treatment is high. The symptom of "stomachache" is more pronounced in females, while "trouble sleeping" is more severe in males. Quality of life plays an essential role in RSS in both genders. Thus, sex needs to be considered when assessing the relationship between RSS and therapeutic effect in depression.

**Supplementary Information:**

The online version contains supplementary material available at 10.1186/s12888-023-04612-3.

## Background

Depression is a common psychiatric disorder characterized by a range of cognitive, affective, and somatic symptoms. It is also characterized by high incidence, recurrence, disability, and suicide rate [1]. Although the optimal treatment goal for patients with depression should be complete remission of symptoms and return to the premorbid functional level, about one-third of patients with depression experience only partial remission and sustained residual symptoms after maintenance treatment [2]. Residual symptoms are often defined as subthreshold depressive symptoms that persist at the end of treatment [3]. The most common residual symptoms can generally be divided into two main categories, depressive symptoms that are not completely relieved, and non-depressive mood symptoms that are dominated by residual somatic symptoms (RSS) [4, 5]. Residual symptoms could damage patients' social function [6] and quality of life [7, 8]. When residual symptoms are detected, clinicians must decide what to do with the following treatment stage, such as continuing current treatment, using different mechanisms of action, switching to intra-class agents, and so on [9].

Previous research shows that more than 40% of responders had physical symptoms during long-term antidepressant treatment. The somatic symptoms may be a side effect of antidepressants and one of the most common residual symptoms in patients with depression [10]. Body-related residual symptoms are good predictors of complete remission in patients with depression during follow-up [11]. A research study shows that residual symptoms associated with somatic symptoms at baseline predict relief of depression during follow-up [12]. Studies have shown that patients who do not achieve complete remission, especially those with more severe physical symptoms, have significant damage to health-related quality of life. These shreds of evidence suggest that RSS play a vital role in treating patients with depression.

The incidence of emotional disorders is on the rise in China. Research shows that the rate of depression was 64.7%, and the rate of somatic complaints was 64.9% [13]. Previous studies have found that the incidence of somatic symptoms, especially dizziness, is increasing in the Chinese population and is highly correlated with panic disorders [14]. In addition, a study measuring the incidence of depression, anxiety, and somatic symptoms in outpatients of general hospitals in China found that depression, anxiety, and somatic symptoms were common in these patients, and further research showed that depression was independently associated with somatic symptoms, female participants had a higher risk of somatic symptoms and emotional distress [15]. Therefore, we found that depression has a certain correlation with somatic symptoms of different sex. Sex differences in depression are now widely acknowledged. Epidemiological and clinical studies have shown that women are twice as likely to suffer from depression as men [16]. Most studies have found that men and women have different symptoms of depression. For example, a study showed that women with depression are more likely to experience atypical depressive episodes, which are associated with higher rates of apparent psychomotor retardation, fatigue hypersomnia, and suicide attempts, while men are more likely to experience decreased libido [17]. There has been some evidence for the explanation of sex differences, including hormone action [18], brain structure and function [19], EEG asymmetry-depression hypothesis [20] and so on.

Some pieces of evidence suggest that sex differences in depression are due to sex differences associated with somatic symptoms, such as fatigue, pain, and appetite [21]. Studies have also shown that the overlap with somatic depression almost entirely leads to sex differences in atypical depression [22]. One study further noted that gender differences in reporting depressive symptoms were only slightly stronger for somatic symptoms, with a ratio of 1.38 for women to men [23].In patients with mood disorders, the additional burden imposed by somatic symptoms may have significant consequences, affecting treatment choice and response to depression and clinical monitoring requirements. Somatic depression is more likely to be antidepressant-resistant [24]. Recent studies have shown different pharmacological profiles for refractory depression, highlighting differences in treatment modalities between genders and the benefits of enhancement strategies for women [25]. Therefore, the results suggest that treatment options for depressed patients with somatic symptoms may differ between males and females. Women are more likely to have somatic symptoms [26], but whether there are differences in somatic symptoms between men and women after depression treatment is unclear and needs to be further explored.

Many factors can influence the onset of residual somatic symptoms. For example, the general condition of the patient. Such as age is related to somatic symptoms and health-related anxiety [27]. The average treatment efficacy for functional somatic symptoms was higher among women and those with higher education levels [28]. The dose and duration of medication can also affect the onset of RSS. One study found that inadequate amounts and periods of antidepressant medicines may affect the control of residual somatic symptoms and have a higher relapse rate [29]. Patients who respond or remit after acute phase treatment often have residual symptoms, such as anxiety, depression, sleep problems, fatigue, and cognitive dysfunction. And these residual symptoms may interfere with their somatic symptoms and functioning [30]. As we know, there is also a correlation between quality of life, social functioning, and somatic symptoms. Most studies show that people with depression experience various somatic symptoms that negatively affect social functioning and reduce their quality of life [31]. However, whether the quality of life and social functioning affect the appearance of residual somatic symptoms is rarely explored. It is unclear whether the general condition of the patients mentioned above, the medication treatment condition, and the presence of symptoms after depression treatment make a clear contribution to the occurrence of RSS and whether there are differences in the influencing factors between men and women. This is something that needs further discussion.

Since sex differences play a prominent role in both depression and somatic symptoms. Exploring gender differences will help us to better understand the mechanisms and comorbidities of depression with somatic symptoms. It is of great clinical significance to understand sex difference of RSS because it will affect the treatment method and efficacy. To exclude the influence of the history of depression on the study results, we selected patients with first-episode depression as the object. This study's purpose is as follows: ⑴To explore the sex differences in demographic factors in patients with and without residual symptoms⑵To identify sex differences in RSS⑶To explore the independent influencing factors of RSS in men and women. Based on previous studies, we propose the following main hypotheses: ⑴ There are gender differences in residual somatic symptoms⑵ There are different factors that influence the occurrence of residual somatic symptoms in men and women.

## Methods

### Patient enrollment

This study was a multi-center, cross-sectional survey involving 11 centers, including 5 psychiatric hospitals and 6 general hospitals with psychiatric departments. From September 2014 to July 2015, a total of more than 1500 outpatients were investigated by continuous sampling method, and 982 patients were finally included in this study. All patients were ≥ 18 years old and required to meet the diagnostic criteria of depressive episodes in the International Classification of Diseases-10 (ICD-10) and confirmed by two independent, experienced psychiatrists. According to the standard treatment guidelines, they received antidepressant therapy for 8 to 12 weeks without interruption for more than two weeks(Antidepressants were the main drugs in the treatment of patients, including SSRIs:720 cases,SNRIs:311 cases,NaSSAs:59 cases,SARIs:11 cases, TCAs, etc.:402 cases). A visual analogue scale (VAS) was used to evaluate the improvement of depressive symptoms. Patients were asked to draw a vertical line and ask, "from your point of view, how much depression have you recovered after this treatment?". VAS is a widely used clinical scoring standard, with a score range of 0 to 10 (0 = no change or deterioration and 10 = best of entirely possible recovery) [32, 33]. The VAS depression scale represents a simple, easily implementable instrument suitable for mental health research in typical settings and more extensive population-based studies [34]. Those who think their recovery is more than 50% will be allowed to enter the study. Patients were excluded if diagnosed with generalized anxiety disorder, bipolar disorder, mania, schizoaffective disorder, schizophrenia, or those with mental disorders caused by somatic diseases. The patient's somatic symptoms were related to a typical physical illness. Major ethics and each hospital ethics approved the research protocol. By signing the consent form, all patients agreed to participate in the study.

### Measures

Using the standard data collection tables designed for this study (Supplementary table), we collected the patients' essential demographic and clinical characteristics. Including the patient's age, years of education, family history of psychiatric disorders, age at onset, duration of current episode, duration of drug treatment in current, history of somatic disease. Self-report instruments included the 16-item Quick Inventory of Depressive Symptomatology Self-Report (QIDS-SR_16_), the PHQ-15, SDS, and Q-LES-Q-SF.

**Fig. 1:**
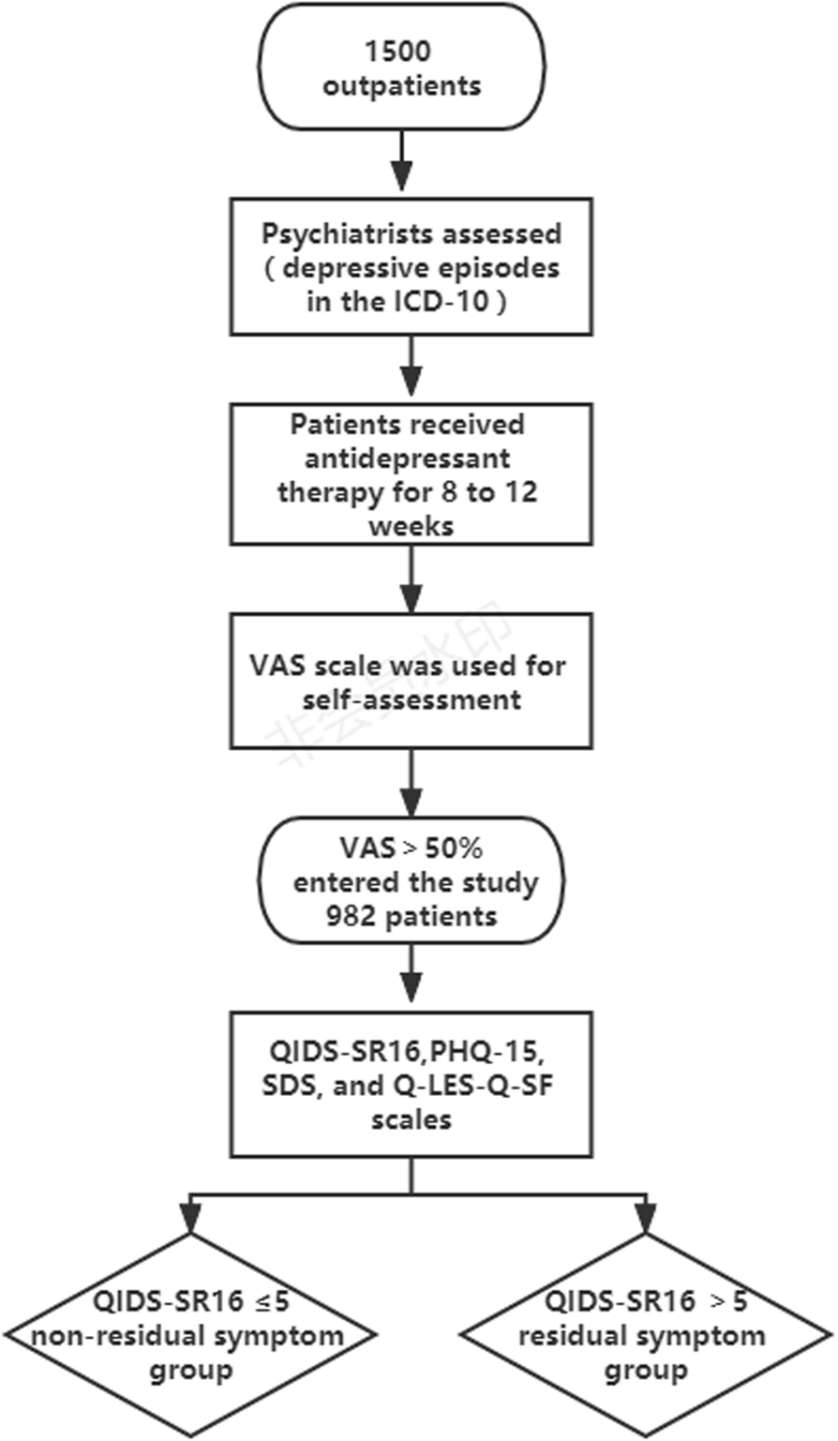
The overall flow chart of the study

QIDS-SR_16_ was used to evaluate the severity of depressive symptoms. QIDS-SR_16_ has good reliability and validity in screening depressive disorders and has been widely used in China [35]. The specificity and sensitivity for major depressive disorder are 0.66 and 0.83, respectively. Cronbach's α is 0.80, indicating good internal consistency [36]. A total score ≤ five was defined as indicating remission [37]. According to the total score of QIDS-SR16, patients were divided into the residual symptoms group and the non-residual symptoms group. The overall flow chart of the study is as follows Fig. 1.

The RSS was assessed by PHQ-15, accounting for more than 90% of physical symptoms reported by outpatients [38]. PHQ-15 is one of the best somatic symptom scales recently confirmed by a critical systematic review [39]. Over the past four weeks, subjects were asked to rate the severity of their somatic symptoms on a scale of 0 ("not bothered at all"), 1 ("a little disturbing"), or 2 ("bothering a lot"). The scores of 0–4, ≥ 5, ≥ 10, ≥ 15 represent minimal, mild, moderate, and severe somatization levels. While the PHQ-15 primarily assesses somatic symptoms over the past four weeks, we mainly asked patients which symptoms you have present during the eight weeks of drug treatment for this experiment to assess residual somatic symptoms.

The SDS was used to evaluate the patients' function [40], and Q-LES-Q-SF was used to assess the patients' enjoyment of life and satisfaction [41].

### Statistics

The statistical analysis system (SAS) software of Windows version 9.2 was used to analyze the data. The demographic and clinical characteristics of the residual symptoms group and the non-residual symptoms group were compared by independent sample chi-square test, t-test, Fisher exact test, and Wilcoxon rank-sum test as appropriate. The sex differences of each item in PHQ-15 were further analyzed by the analysis of ANOVA. By adding the variables into the analysis model as covariables, we can judge whether the variance analysis is significant.

Stepwise multiple Logistic regression analyses were used to determine the independent demographic and related clinical factors of RSS in males and females with residual symptoms. The independent variables included demography (age, education level, history of somatic disease), condition of disease (duration of current episode, duration of drug treatment in the recent episode, family history of psychiatric disorders), a total score of QIDS-SR_16,_ and scores of each item, total score of SDS and Q-LES-Q-SF. And before conducting the regression analysis, we performed a covariance diagnosis to remove indicators with variance inflation factors (VIF) greater than 10. The statistical significance for all tests was set at *P* < 0.05 of two-tailed tests.

## Results

### Comparison of demographic, clinical information, and RSS severity

There were 449 cases (45.72%) in the residual symptoms group and 533 cases (54.28%) in the non-residual symptoms group. The primary demographic and clinical features are shown in Table 1. Compared with the non-residual symptoms group, the residual symptoms group patients were younger and younger at the time of onset (*P* = 0.0197). Patients in the residual symptoms group had a higher frequency of comorbid physical diseases (21.16% vs. 16.14%, *P* = 0.0431). The total score of PHQ-15 and SDS in the residual symptoms group was higher than that in the non-residual symptoms group, while the total score of Q-LES-Q-SF was lower(*P* < 0.0001).

**Table 1:** Overall comparison between groups and comparison of gender differences within groups

Items		Non-residual symptoms group (QIDS ≤ 5)			Residual symptoms group (QIDS > 5)			*t*/X^2^	*P*
		Total	Female	Male	Total	Female	Male		
*n*(%)		533	334(62.66)	199(37.34)	449	285(63.47)	164(36.53)		
Age(year)		43.96 ± 14.13	44.71 ± 13.70	42.70 ± 14.78	41.79 ± 14.92	43.24 ± 14.81	39.27 ± 14.81^++^	2.34	0.0197^*^
Education (year)		12.09 ± 3.71	11.80 ± 3.80	12.57 ± 3.51^+^	12.42 ± 3.77	12.09 ± 3.86	12.99 ± 3.54^+^	-1.39	0.1651
Family history of psychiatric disorders [*n*(%)]		53(9.94)	39(11.68)	14(7.04)	44(9.80)	25(8.77)	19(11.59)	0.01	0.9399
Age at onset (year)		43.96 ± 14.13	44.71 ± 13.70	42.70 ± 14.78	41.79 ± 14.92	43.24 ± 14.81	39.27 ± 14.81^++^	2.34	0.0197^*^
Duration of current episode [*n*(%)]	≤ 3 months	214(40.15)	130(38.92)	84(42.21)	206(45.88)	126(44.21)	80(38.83)	3.27	0.0706
	> 3 months	319(59.85)	204(61.08)	115(57.79)	243(54.12)	159(55.79)	84(34.57)		
Duration of drug treatment in current episode(week)		10.25 ± 1.60	10.19 ± 1.61	10.33 ± 1.58	10.10 ± 1.60	10.18 ± 1.58	9.95 ± 1.62	1.45	0.1481
History of somatic disease [*n*(%)]	with	86(16.14)	58(17.37)	28(14.07)	95(21.16)	65(22.81)	30(18.29)	4.09	0.0431^*^
	without	447(83.86)	276(82.63)	171(85.93)	354(78.84)	220(77.19)	134(81.71)		
PHQ total score		3.16 ± 2.78	3.33 ± 2.93	2.86 ± 2.48	7.26 ± 4.29	7.33 ± 4.37	7.15 ± 4.16		< 0.0001^**^
QIDS total score		2.80 ± 1.63	2.77 ± 1.62	2.85 ± 1.64	9.65 ± 3.89	9.39 ± 3.65	10.12 ± 4.24		< 0.0001^**^
SDS total score		3.57 ± 3.72	3.55 ± 3.57	3.61 ± 3.95	10.18 ± 7.20	9.80 ± 7.38	10.86 ± 6.86		< 0.0001^**^
Q-LES-Q-SF total score (first 14 items)		48.67 ± 6.49	48.28 ± 6.19	49.32 ± 6.93	40.72 ± 6.73	41.18 ± 7.02	39.93 ± 6.14^+^	18.80	< 0.0001^**^

^*^ indicates the comparison between the residual symptoms group and the non-residual symptoms: ^*^P < 0.05, ^**^P < 0.01

^+^ indicates the comparison between males and females in the residual symptoms group or the non-residual symptoms: ^+^*P* < 0.05, ^++^*P* < 0.01

Further covariance analysis showed that after controlling other factors and adding sex, age, comorbid somatic disease, residual symptoms, and duration of the current episode into the equation, only residual symptoms had a statistically significant effect on the total score of PHQ-15 (*P* < 0.0001). In the residual symptoms group, the age and age at onset in females were higher than in males, and the total score of Q-LES-Q-SF was higher in females than in males (*P* < 0.05). The education years of males in the residual symptoms group and the non-residual symptoms group were longer than females (*P* < 0.05).RSS frequency in the residual symptoms group was significantly higher than that in the non-residual symptoms group (73.50% vs. 23.64%, *P* < 0.0001). There was a significant difference in the distribution of PHQ-15 severity between the two groups (*P* = 0.0006). See Table 2.

**Table 2:** Comparison of the severity of residual somatic symptoms between the two groups

Item		Non-residual symptoms group	Residual symptoms group	χ^2^	*P*
Residual somatic symptoms [*n*(%)]	With (≥ 5)	126 (23.64)	330 (73.50)	243.5481	< 0.0001
	Without (< 5)	407 (76.36)	119 (26.50)		
PHQ total score [*n*(%)]	mild(5 ~ 9 scores)	107 (84.92)	221 (66.97)	14.7642	0.0006
	middle(10 ~ 14 scores)	14 (11.11)	73 (22.12)		
	sever(≥ 15 scores)	5 (3.97)	36 (10.91)		

### Sex differences of RSS in the residual symptoms group

For patients with residual symptoms, we further explored the occurrence of RSS and gender differences. Patients with a total PHQ-15 score ≥ 5 were considered to have residual somatic symptoms and were further analyzed. The top five RSS in the residual symptoms group were feeling tired or having low energy (76.39%), trouble sleeping (67.71%), headache (55.01%), constipation, loose bowels, or diarrhea (51.22%), and feeling your heart pound or race (50.33%). Table 3 showed sex differences in each item and the total scores of PHQ-15 in patients with residual symptoms. The symptoms of "stomach pain" in females were more severe than those in males, while the symptoms of "trouble sleeping" in males were more stringent than those in the female (*P* < 0.05). When the effects of age, comorbid somatic disease, QIDS-SR_16_ total score, the duration of drug treatment in the current episode were added into the ANOVA as covariates, there were still significant differences between males and females (*P* = 0.0032).

**Table 3:** Gender differences in residual somatic symptoms of patients with residual symptoms

Item	Female	Male	Total	*t*	*P*	95% confidence interval for EXP(B)		
						Exp(B)	Lower	Upper
Stomach pain	0.41 ± 0.49	0.31 ± 0.46	0.37 ± 0.48	2.10	0.0359	0.0996	0.00659	0.1925
Back pain	0.36 ± 0.48	0.31 ± 0.46	0.34 ±	1.01	0.3136	0.0469	-0.0445	0.1383
Pain in your arms, legs or joints	0.35 ± 0.48	0.40 ± 0.49	0.37 ± 0.48	-0.89	0.3763	-0.0420	-0.1351	0.0512
Menstrual cramps or other problems with your periods (Female only)	0.27 ± 0.44	NA	0.27 ± 0.44	-	-	-		
Headaches	0.57 ± 0.50	0.51 ± 0.50	0.55 ± 0.50	1.22	0.2214	0.0597	-0.0361	0.1556
Chest pain	0.25 ± 0.43	0.22 ± 0.42	0.24 ± 0.43	0.71	0.4794	0.0296	-0.0526	0.1118
Dizziness	0.45 ± 0.50	0.49 ± 0.50	0.46 ± 0.50	-0.79	0.4298	-0.0387	-0.1349	0.0575
Fainting spells	0.07 ± 0.26	0.09 ± 0.29	0.08 ± 0.27	-0.67	0.5052	-0.0178	-0.0702	0.0346
Feeling your heart pound or race	0.52 ± 0.50	0.48 ± 0.50	0.50 ± 0.50	0.89	0.3738	0.0437	-0.0527	0.1401
Shortness of breath	0.39 ± 0.49	0.35 ± 0.48	0.38 ± 0.49	0.96	0.3399	0.0454	-0.0480	0.1389
Pain or problems during sexual intercourse	0.12 ± 0.32	0.10 ± 0.30	0.11 ± 0.31	0.60	0.5519	0.0182	-0.0419	0.0784
Constipation, loose bowels, or diarrhea	0.50 ± 0.50	0.53 ± 0.50	0.51 ± 0.50	-0.59	0.5586	-0.0287	-0.1252	0.0677
Nausea, gas, or indigestion	0.44 ± 0.50	0.41 ± 0.49	0.43 ± 0.50	0.62	0.5364	0.0301	-0.0654	0.1255
Feeling tired or having low energy	0.75 ± 0.43	0.79 ± 0.41	0.76 ± 0.43	-0.86	0.3921	-0.0357	-0.1176	0.0462
Trouble sleeping	0.64 ± 0.48	0.74 ± 0.44	0.68 ± 0.47	-2.31	0.0216	-0.1053	-0.1950	-0.0156
Total scores	7.33 ± 4.37	7.15 ± 4.16	7.27 ± 4.29	0.44	0.6570	0.1870	-0.6401	1.0141

### Multiple regression analysis of PHQ-15 scores in males with residual symptoms

A multiple regression model was established using the PHQ-15 score as a dependent variable, age, family history of psychiatric disorders, QIDS-SR_16_ scale scores, and other meaningful variables as independent variables. The results demonstrated that the total score of QIDS-SR_16_, the 12th item of QIDS-SR_16,_ and the total score of Q-LES-Q-SF were independent influencing factors for RSS. Furthermore, the RSS of patients with depression after acute phase treatment were negatively correlated with the total score of QIDS-SR_16_ (B = -0.2871,* P* = 0.0133) and positively associated with the 12th item of QIDS-SR_16_ of suicidal ideation (B = -2.2493,* P* = 0.0019), and the total score of Q-LES-Q-SF (B = -0.0938,* P* = 0.0026), see Table 4.

**Table 4:** Multiple regression analysis of PHQ scores in male with residual symptoms

Variables	Regression coefficient	WALD 2	*T*	*P*	Standardized coefficient
Age	0.0226	0.0200	1.2706	0.2597	0.1858
Education (year)	0.0890	0.0728	1.4950	0.2214	0.1738
Family history of psychiatric disorders	0.2510	0.7632	0.1082	0.7422	0.0439
Duration of current episode (≤ 3 months VS. > 3 months)	-0.0735	0.1030	0.5091	0.4755	-0.0894
Duration of drug treatment in current episode (week)	-0.0394	0.1278	0.0950	0.7579	-0.0350
History of somatic disease(with vs without)	0.3502	0.6693	0.2738	0.6008	0.0746
QIDS total scores	-0.2871	0.1159	6.1317	0.0133	-0.6678
QIDS item 1	-0.0247	0.2438	0.0102	0.9194	-0.0135
QIDS item 2	-0.1438	0.2527	0.3237	0.5694	-0.0819
QIDS item 3	0.2407	0.2549	0.8917	0.3450	0.1394
QIDS item 4	0.2613	0.3119	0.7015	0.4023	0.1187
QIDS item 5	-0.3110	0.3115	0.9968	0.3181	-0.1747
QIDS item 6	-0.6578	0.3548	3.4360	0.0638	-0.2599
QIDS item 7	0.1229	0.4167	0.0870	0.7681	0.0467
QIDS item 8	-0.4768	0.2819	2.8617	0.0907	-0.2101
QIDS item 9	-0.5472	0.3412	2.5718	0.1088	-0.2313
QIDS item 10	0.2245	0.9205	0.0595	0.8073	0.0428
QIDS item 11	0.3727	0.5826	0.4093	0.5223	0.1010
QIDS item 12	2.2493	0.7251	9.6233	0.0019	0.5855
QIDS item 13	-0.0865	0.7372	0.0138	0.9066	-0.0209
QIDS item 14	-0.3349	0.8321	0.1620	0.6873	-0.0749
QIDS item 15	0.1654	0.7746	0.0456	0.8309	0.0406
QIDS item 16	-0.8518	0.5256	2.6267	0.1051	-0.2355
SDS total scores	-0.0120	0.0467	0.0656	0.7979	-0.0446
Q-LES-Q-SF total scores	0.0938	0.0312	9.0548	0.0026	0.3174

### Multiple regression analysis of PHQ-15 scores in females with residual symptoms

After analyzing related factors, a multiple regression model was established to explore the independent influencing factors for RSS in females. PHQ-15 score was regarded as a dependent variable, while age, education years, duration of drug treatment in the current episode, QIDS-SR_16_ scores, duration of drug treatment in current episode, history of somatic disease, SDS total scores, and Q-LES-Q-SF total scores were selected as meaningful variables. It was found that the QIDS-SR_16_ item 1, QIDS-SR_16_ item 14, SDS total score, and the total score Q-LES-Q-SF played independent influencing factors for RSS. The total score of PHQ-15 was negatively correlated with the total score of sleep-onset insomnia of QIDS-SR_16_ item 1 (B = -0.3583,* P* = 0.0450), QIDS-SR_16_ item 14 of energy level (B = -1.1872,* P* = 0.0228), SDS total score (B = -0.0575,* P* = 0.0313), and positively correlated with the total score Q-LES-Q-SF (B = 0.0956,* P* < 0.0001), see Table 5.

**Table 5:** Multiple regression analysis of PHQ scores in female with residual symptoms

Variables	Regression coefficient	Wald 2	*T*	*P*	Standardized coefficient
Age	0.0133	0.0129	1.0647	0.3021	0.1078
Education (year)	0.0693	0.0464	2.2304	0.1353	0.1475
Family history of psychiatric disorders	-0.2479	0.5809	0.1821	0.6696	-0.0394
Duration of current episode (≤ 3 months VS. > 3 months)	-0.0789	0.0761	1.0747	0.2999	-0.0896
Duration of drug treatment in current episode (week)	-0.0953	0.0913	1.0901	0.2964	-0.0832
History of somatic disease(with vs without)	0.2605	0.4045	0.4147	0.5196	0.0605
QIDS total scores	0.0925	0.0979	0.8922	0.3449	0.1865
QIDS item 1	-0.3583	0.1788	4.0180	0.0450	-0.2039
QIDS item 2	-0.1069	0.1835	0.3391	0.5603	-0.0567
QIDS item 3	-0.2920	0.1833	2.5380	0.1111	-0.1544
QIDS item 4	0.1321	0.2352	0.3151	0.5745	0.0561
QIDS item 5	-0.3613	0.2681	1.8165	0.1777	-0.1755
QIDS item 6	-0.0432	0.3013	0.0206	0.8860	-0.0142
QIDS item 7	-0.4338	0.2701	2.5796	0.1082	-0.1631
QIDS item 8	-0.3627	0.2692	1.8150	0.1779	-0.1321
QIDS item 9	-0.1214	0.2203	0.3039	0.5814	-0.0599
QIDS item 10	-0.3229	0.4545	0.5047	0.4774	-0.0724
QIDS item 11	-0.5668	0.4315	1.7252	0.1890	-0.1470
QIDS item 12	0.3256	0.3908	0.6942	0.4047	0.0834
QIDS item 13	0.0892	0.4638	0.0370	0.8475	0.0213
QIDS item 14	-1.1872	0.5214	5.1835	0.0228	-0.2846
QIDS item 15	-0.4829	0.4065	1.4109	0.2349	-0.1275
QIDS item 16	0.2309	0.3444	0.4494	0.5026	0.0633
SDS total scores	-0.0575	0.0267	4.6373	0.0313	-0.2332
Q-LES-Q-SF total scores	0.0956	0.0222	18.4875	< .0001	0.3687

## Discussion

To our best knowledge, this is the first study to explore sex differences and related factors in RSS in Chinese patients with FED after acute stage treatment. We mainly found that the age and age of onset of females were older than males for patients with residual symptoms. Males had more years of education than females. From the perspective of RSS, the "stomach pain" of females was more severe than males, while males showed more severe "trouble sleeping." Some residual depressive symptoms were associated with the occurrence of RSS. Multiple regression analysis showed that the total Q-LES-Q-SF score was an independent influencing factor of RSS in both males and females, while the total SDS score only affected female RSS.

Our study found that people younger and younger at the time of onset were more likely to have residual symptoms. The same result is that early-onset depression has higher levels of residual symptoms over time [42]. This can be explained by the "a stage of illness" hypothesis: the early-onset group has a shorter remission period and may develop further during the progression of depression [43]. We also found that patients with residual symptoms had a lower quality of life and a more significant impact on functional impairment. There is evidence that residual depression is thought to have as many functional effects as acute diseases [44]. Residual symptoms lead to reduced quality of life [7]. All these results are consistent with our data. We also found that patients with previous physical diseases were more likely to have residual symptoms. A previous study showed that physical symptoms at baseline are associated with remission of depression [11], partly supporting our findings. In addition, in some cross-cultural somatic symptom-related studies, Chinese population depression studies have shown quite prominent somatic symptoms [45]. 76% of Chinese-American primary care patients with depression reported complaints centered on physical symptoms [46]. In a comparison of outpatient psychiatric samples with depression in Toronto (Caucasian) and China, the results of spontaneous problem reporting and structured clinical interviews showed that Chinese patients had more physical symptoms and fewer psychological symptoms [47]. The above studies also directly or indirectly support our findings that some residual depressive symptoms are associated with the development of RSS. Perhaps in future comparative studies with larger samples, we can further explore whether the residual physical symptoms of different genders are correlated with different races and cultures.

Although the primary goal of depression treatment is clinical recovery, many patients still have residual symptoms after treatment with antidepressants. Among the residual symptoms, RSS is one of the most common ones. A post-hoc analysis showed that the prevalence rate of residual symptoms was 41% for somatic symptoms [48]. In the study of Paykel et al., it has been reported that a typical combination of emotional and physical symptoms forms residual symptoms [49]. Our study found that patients with residual symptoms were more likely to have RSS. At the same time, the RSS would be more serious. A study focused on residual painful physical symptoms (PPS) shows that the prevalence of at least moderate PPS in patients with partial remission is higher than that in patients with complete remission [5], which is consistent with our findings.

We found that females had more severe stomachache than males. A meta-analysis shows that functional abdominal pain disorders are more likely to occur in girls and are associated with depressive disorders in children [50]. Although the age is not consistent with our experiment, it still confirms the fact that females are more likely to develop abdominal pain. It is well known that pain is mainly related to the degree of inflammation. The results of an animal experiment show that gastric inflammation leads to anxiety and depression-like behavior in female rats rather than male rats through the neuroendocrine (HPA axis) pathway, suggesting that gastrointestinal inflammation can induce psychological and behavioral changes through inflammatory gastrointestinal-to-brain signals in a sex-related manner [51]. We must recognize the anti-inflammatory effects of androgens and the pro-inflammatory effects of estrogen [52]. These may be the basis of inflammation and sex differences in MDD and may explain these differences [53], as we found. Studies have also shown that psychogenic somatoform symptoms require approximately 7–11 weeks to improve somatic symptoms. Previous reports have shown that in depressed patients, stomach pain stabilizes earlier than improvement in depressive symptoms, and improvement in somatic symptoms is concentrated within the first month of treatment and then essentially plateaus [54]. The results suggest that we should pay attention to somatic symptoms that are not relieved. And the study showed that in most depressed patients with psychogenic somatic symptoms whose symptoms have been improved, the serum 3-Hydroxybutyrate (3HB) levels were initially (pre-treatment) elevated and decreased after treatment with antidepressants [55]. Plasma 3HB levels were found to rise more in women than in men [56]. This conclusion also explains why women are more likely to have stomach pain symptoms after treatment in the acute phase.

At the same time, males had more "trouble sleeping" than females. Carmona's study found that anxiety and sleep disorders in men and the severity of depression in women determine their functional disabilities, suggesting that sleep problems may significantly impact men [57]. Other studies have shown the opposite results [58]. This may be related to the different samples and time of observation. Although the exact mechanism of sex differences in insomnia is unknown, we have suggested some potential mechanisms for gender differences in insomnia. Men were found to be more burdened with other risk factors for insomnia, such as smoking, snoring, and alcohol consumption. Another possible explanation for the association with sleep–wake regulation is that females have lower homeostatic drive than males [59]. The electroencephalographic (EEG) slow-wave activity during NREM sleep (an indicator of the homeostatic drive of sleep requirements) shows that females exhibit more slow-wave activity than males at baseline and after sleep deprivation. This observation is consistent with the objective measure that women objectively sleep better than men. The above results also suggest that men may sleep worse [60].

We also found that quality of life was an independent factor affecting RSS in both males and females. A study shows that patients with somatic symptom disorder are associated with depression and quality of life [61]. Currently, most of the experiments are focused on the impact of somatic symptoms on quality of life. For example, one study showed that patients with unremitting MDD, especially those with more severe somatic symptoms, exhibit significant quality of life impairment and more clinical symptoms, demonstrating the importance of achieving remission in treating MDD [62]. Our research shows that quality of life can also affect the occurrence of residual somatic symptoms. However, follow-up studies must further elucidate the relationship between the above two. Another study of patients with complete remission of depression showed that patients with impaired social function had higher levels of somatization than healthy controls, suggesting that social function may have an impact on somatization [63]. This study supports our finding that the female's social function is an independent factor affecting somatic symptoms. We also discovered some depressive symptoms were closely related to the occurrence of somatic symptoms, such as total QIDS-SR16 scores, death, suicidal ideation, sleep problems, and energy. Some studies support our view, such as the somatic symptoms of patients with a first-episode major depressive disorder are closely related to suicidal ideation [64]; Persistent depressive disorder was independently associated with more severe somatic symptoms [65]. In a longitudinal study of aging, the authors point out that sleep problems, depressive symptoms, and their combination are differently associated with a physical illness that occurs six years later [66]. All these suggest that clinicians should pay attention to patients' somatic symptoms in practice.

Our research has several limitations. First, QIDS-SR_16_ and PHQ-15 are self-rating scales. Many factors may affect doctors' and patients' consensus on the severity of subjective symptoms, which can be further improved by adding observer-rating scales in future studies. Both QIDS-16 and PHQ-15 do have similar items assessed, such as energy and sleep, which also prompted us to use multiple assessments for somatic symptoms in the follow-up study to try to uncover things that are different from those not repeated in the depression rating scale. In the present experiment, we collected somatic diseases and asked about somatic symptoms due to somatic disorders. We did not include patients whose somatic symptoms were caused by somatic diseases. However, this was the content of the clinical interview, and no particular form was designated to record the relationship between patients' detailed somatic symptoms and diseases. We will select the corresponding format for statistics in future experiments. Secondly, the samples should continue to be collected, such as inpatients and community patients. Third, more variables should be managed, such as personality characteristics, family environment, drug use of patients with pre-illness physical diseases, smoking and other influencing factors. Fourth, the treatment time, treatment methods, and the number of depressive episodes can be further controlled to improve the accuracy of the results. Fifth, make a more detailed classification study on the causes of residual symptoms. Finally, with the limitations of a typical cross-sectional study, this study cannot determine the causal relationship between various factors and somatic symptoms. For example, for quality of life and RSS, only correlation conclusions can be drawn now, and no causality can be determined. Follow-up causality validation requires further confirmation in follow-up studies, providing new directions for our subsequent studies. Future studies should include whether there are sex differences in the long-term treatment of somatic symptoms in patients with depression.

## Conclusion

Our study found that patients with residual symptoms had a higher proportion of RSS. There are sex differences in RSS, especially in female patients with "stomachache" and male patients with "trouble sleeping". Some depressive symptoms and patients' social function have an influence on the occurrence of RSS. Quality of life is an independent influencing factor of RSS in both men and women, and attention should be paid to the improvement of patients' quality of life. This study also suggests that more attention should be paid to somatic symptoms in the treatment of patients with depression.

## Supplementary Information

**Additional file 1. **Supplementary table

## Data Availability

The datasets used and/or analysed during the current study are available from the corresponding author on reasonable request.

## Abbreviations

RSS: Residual somatic symptoms
FED: First-episode depression
PHQ-15: Patient health questionnaire-15
SDS: Sheehan disability scale
Q-LES-Q-SF: Quality of life enjoyment and satisfaction questionnaire-short form
VAS: Visual analogue scale
QIDS-SR_16_: 16-Item quick inventory of depressive symptomatology self-report
PPS: Painful physical symptoms
