# Supplementary material for: Sex differences in residual somatic symptoms in patients with first-episode depression after acute-phase treatment
Source: BMC Psychiatry. 2023 Feb 22;23:119. doi: 10.1186/s12888-023-04612-3 (PMC9948378; doi:10.1186/s12888-023-04612-3)
Supplement: Supplementary file 1 — Additional file 1. Supplementary table [file 12888_2023_4612_MOESM1_ESM.docx]

1. General information
   1. Date of visit __ __ __ __Year__ __Month__ __Day
   2. Gender 🗆Female 🗆Male
   3. Patient's birth year and month __ __ __ __Year__ __Month
   4. Number of years of patient education
2. History of depression
   1. Duration of current episode
   2. Whether this depressive episode is a first episode
   3. Time already receiving medication for this depressive episode __ __weeks
3. Somatic Diseases
   1. 🗆With 🗆Without
   2. Specific somatic disease diagnosis

| Diagnosis1: | 🗆Cured 🗆Currently suffering from |
| --- | --- |
| Diagnosis2: | 🗆Cured 🗆Currently suffering from |
| Diagnosis3: | 🗆Cured 🗆Currently suffering from |
| Diagnosis4: | 🗆Cured 🗆Currently suffering from |
| Diagnosis5: | 🗆Cured 🗆Currently suffering from |

1. Medication treatment since this depressive episode

4.1 For all medications used (including those currently being used) since the current depressive episode, please fill out one form for each medication by medication category.

| Drug |  |
| --- | --- |
| Total duration of use of the drug since this depressive episode (days) |  |
| Maximum dose of the drug ever used after the current depressive episode(mg/d) |  |
| Duration of use of the maximum dose(days) |  |
| Longest used dose(mg/d) |  |
| Is the drug currently being used | 🗆Yes 🗆No |
| Current dose used |  |

4.2 Has the antidepressant or antipsychotic been changed since the current depressive episode? If yes, please indicate the specific medication changes.

1. Family history of depression

🗆Yes, Relationship with patients:_______________ 🗆No
